# Extruded canine diets containing primarily peas in contrast to those containing lamb and chicken meal are at higher risk of mold and mycotoxin contamination when treated similarly: An observational study

**DOI:** 10.1002/fsn3.4277

**Published:** 2024-06-17

**Authors:** Michelina Crosbie, Julia G. Pezzali, Leslie Hancock‐Monroe, Preston R. Buff, Anna K. Shoveller

**Affiliations:** ^1^ Department of Animal Biosciences University of Guelph Guelph Ontario Canada; ^2^ The J.M. Smucker Co. Orrville Ohio USA; ^3^ Present address: Department of Grain Science and Industry Kanas State University Manhattan Kanas USA; ^4^ Present address: Hill's Pet Nutrition, Inc. Topeka Kanas USA; ^5^ Present address: Post Holdings, Inc. St. Louis Missouri USA

**Keywords:** extruded dog food, grain‐free pet food, pet food storage, pet nutrition

## Abstract

Three extruded dog diets were created for a nutritional study with different primary protein sources (BAS: lamb meal (LM) and deboned lamb (DL); CHK: chicken meal, LM, and DL; PEA: dried ground pea, LM, and DL). All diets were processed using the same single‐screw extruder, shipped from the processing facility on the same day, and transported under the same conditions in January 2021. After 8 months of storage in a temperature and humidity‐controlled room in September 2021, only the PEA diet was molded upon inspection. Mold and mycotoxin analysis of all diets was conducted in both September 2021 and at expiry in January 2022, which confirmed mold and mycotoxin contamination to some degree in all diets and most pronounced in the PEA diet across both timepoints. Nutrient analysis of all diets was conducted at production and 2 months post‐expiry in March 2022. As expected, fatty acid and vitamin contents of all diets decreased between sampling timepoints, and amino acid contents generally remained stable. Methionine decreased by 14% in CHK, cystine decreased by 15% and 20% in CHK and PEA, respectively, tyrosine decreased by 30%, 25%, and 27% across BAS, CHK, and PEA, respectively, and taurine decreased by 50%, 42%, and 55% across BAS, CHK, and PEA, respectively. Inaccurate measurement of the PEA diet moisture content post‐production likely led to mold development which may also negatively impact the availability of nutrients and could put dogs at risk for mycotoxicosis and nutrient deficiencies if not closely monitored, but controlled studies are required.

## Graphical abstract

Dog diets containing high‐inclusion levels of peas require greater steam and moisture input during extrusion which may lead to increased risk of mold and mycotoxin contamination during storage if moisture contents of finished products are inaccurately measured. Additionally, mold and mycotoxin contamination may negatively impact availability of nutrients outside of normal shelf‐life losses.

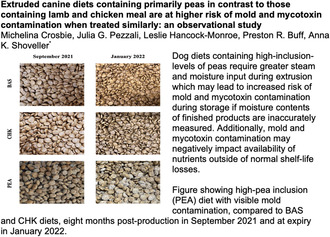

## 1 INTRODUCTION

It is estimated that approximately 75% of pet dogs consume extruded pet food with grain‐free diets (GF; containing primarily peas, pulses, and tubers in place of grains) making up more than 40% of pet foods available in the United States alone (Morelli et al., 2021; Plantz, 2017). Further, it is estimated that many commercial GF diets contain greater than 40% inclusion of pulses (Mansilla et al., 2019). Regardless of the major carbohydrate source utilized in dry pet foods, safety of finished products for both pets and humans is an important factor to evaluate for all diets. Therefore, it is important to consider the potential for mold and mycotoxin contamination in dry pet foods to ensure their shelf‐life and overall food safety.

Mold, and subsequent mycotoxin contamination, in crops can occur during preharvest, harvest, processing, transportation, and storage (Mobashar, 2023). Many surveys have explored the prevalence of mycotoxins in pet foods that have been identified in pet foods worldwide (Bastianello et al., 1987; FDA, 2005; Garland and Reagor, 2001; Stenske et al., 2006). Aflatoxins (particularly B_1_), ochratoxins (particularly ochratoxin A), and *Fusarium* mycotoxins (including deoxynivalenol; **DON**) have been widely found in pet foods at varying levels (Leung et al., 2006). Grain substitutes such as peas and tubers have a higher swelling power and water‐holding capacity than cereal ingredients, requiring more water during extrusion to maintain similar physical characteristics in the finished kibble product (Gujska et al., 1994; Pezzali & Aldrich, 2019). Moisture content accounts for the amount of water present in a feed in contrast to water activity (**a**_**w**_) that accounts for the state of that water, or free water, and indicates the propensity for mold growth to occur if stored at improper temperature and relative humidity conditions (Garces‐Vega et al., 2019). Therefore, this additional moisture may lead to an increased risk of mold and mycotoxin contamination without proper storage of finished products by consumers (Magan et al., 2003). While molds are susceptible to high heat during processing of extruded pet food, mycotoxins are generally chemically and thermally stable and therefore persist in finished kibble after processing (Leung et al., 2006). Mycotoxins are low‐molecular‐weight compounds, undetectable by consumers, and put pets at risk for mycotoxicosis if pet food companies do not conduct robust testing of raw ingredients and finished products (Leung et al., 2006). Further, if the finished kibble product maintains a higher a_w_ post‐extrusion this may put it at higher risk of molding and subsequent mycotoxin production, especially if not properly stored (Garces‐Vega et al., 2019; Magan et al., 2003). Fungal contamination can also lead to a reduction in palatability and nutrients. While the loss of nutrients over time, such as fatty acids and vitamins, has been more extensively studied due to factors like oxidation, exposure to UV, and changes in pH (Coelho, 1991; Hołda & Głogowski, 2016), there has been limited reporting on the impact of mold on the concentration of nutrients in dry pet food over time.

Thus, the objective of this observational study was to evaluate changes in nutrient content of three experimental extruded diets for dogs, produced with different protein and carbohydrate sources, which developed mold 8 months after production.

## 2 MATERIALS AND METHODS

### 2.1 Experimental diets

Three test diets were formulated to conduct nutritional research determining the bioavailability of methionine (**Met**) in large‐breed adult dogs (Table 1). These diets were experimental, not commercially available and included a lamb‐based diet (**BAS**), a chicken‐based diet (**CHK**), and a pea‐based diet (**PEA**) where these protein‐containing ingredients were added at the expense of corn starch to make diets isoenergetic and formulated to predetermined Met content targets, but not crude protein contents (Table 2). Calculated metabolizable energies of the BAS, CHK, and PEA diets were 3433, 3403, and 3329 kcal/kg on a dry matter basis, respectively. Crude protein contents of the BAS, CHK, and PEA diets were 16.26%, 21.18%, and 25.67% on a dry matter basis, respectively. All other nutritional contents (except for fiber) were formulated to be held constant across all three diets. Fiber was not held constant due to the inherently high‐fiber content of peas, and this was not within the scope of the original study.

**TABLE 1:** Ingredient composition of experimental diets on as‐fed (%) basis.

	Diet^a^		
Ingredient, %	BAS	CHK	PEA
Peas, ground dried	–	–	44.14
Chicken meal	–	7.11	–
Lamb meal	15.87	15.00	15.00
Lamb, deboned	10.00	9.50	9.50
Corn starch	42.49	36.90	–
Barley, pearled	20.00	20.00	20.00
Chicken fat	5.61	5.58	6.77
Beet pulp	3.00	3.00	2.39
Animal digest	1.00	1.00	1.00
Potassium chloride	0.80	0.71	0.05
Salt	0.50	0.50	0.50
Vitamin mix^b^	0.26	0.26	0.26
Mineral mix^c^	0.25	0.23	0.21
Choline chloride	0.16	0.14	0.13
Naturox, dry	0.03	0.03	0.03
Naturox, liquid	0.03	0.03	0.03

Abbreviations: BAS, basal diet containing lamb as the primary protein source; CHK, chicken diet containing chicken and lamb as the primary protein sources; PEA, pea diet containing peas and lamb as the primary protein sources.

^a^ All diets produced by The J.M. Smucker Co. (Orrville, Ohio, USA) in their pilot plant in Topeka, Kansas, USA.

^b^ Proprietary blend containing: Vitamin E Supplement, l‐ascorbyl 2‐polyphophate, Niacin Supplement, Vitamin A Supplement, Thiamine Mononitrate, Pantothenic Acid, Riboflavin Supplement, pyridoxine Hydrochloride, Vitamin B12 Supplement, Folic Acid, Biotin, Vitamin D3 Supplement.

^c^ Proprietary blend containing: Potassium Chloride, Ferrous Sulfate, Zinc Sulfate, Copper Sulfate, Sodium Selenite, Manganese Sulfate, Calcium Iodate.

**TABLE 2:** Analyzed nutrient contents of experimental diets on a dry matter basis (%) in January 2021.

Item	Diet^a^		
	BAS	CHK	PEA
ME, kcal/kg as‐fed^b^	3433	3403	3329
Moisture, %	8.40	10.30	12.30
Dry Matter, %	91.62	89.70	87.70
Crude protein, %	16.26	21.18	25.67
Crude fat, %	10.8	12.3	13.8
Crude fiber, %	1.6	1.7	4.4
Total dietary fiber, % as‐fed	6.6	4.9	12.2
Soluble dietary fiber, % as‐fed	1.8	2.2	2.0
Insoluble dietary fiber, % as‐fed	4.9	2.7	10.2
NFE, g/100 g as‐fed^c^	59.2	51.5	43.2
Ash, %	6.8	7.5	6.8

Abbreviations: BAS, basal diet containing lamb as the primary protein source; CHK, chicken diet containing chicken and lamb as the primary protein sources; PEA, pea diet containing peas and lamb as the primary protein sources.

^a^ All diets produced by The J.M. Smucker Co. (Orrville, Ohio, USA) in their pilot plant in Topeka, Kansas, USA.

^b^ ME calculated using modified Atwater calculation.

^c^ NFE calculated using NFE, g/100 g = 100−(moisture + crude protein + crude fat + crude fiber + ash).

### 2.2 Processing and storage of diets

All diets were processed in January 2021 using a single‐screw pilot extruder (model E525, Extru‐Tech, Inc., Sabetha, KS) at The J.M. Smucker Co. (Orrville, Ohio, USA) pilot processing plant in Topeka, Kansas, USA, on the same day using the same ingredient batches (Table 3). Dry ingredients for all batches of all three diets were blended for 5 min using a 1.2 mm mill screen size. In all diets, the deboned lamb was added as a slurry in the preconditioner at a rate of approximately 87 kg/h. The extruder profile used die restriction only. A circular die (6.0 mm diameter) with four inserts was used to produce standard size kibble for dogs. Similar wet bulk density out of the extruder (~368 g/L) was set as the targeted parameter during production, and adjustments of processing conditions were allowed to keep product wet bulk density as specified above. All three diets used the same raw material dry feed rate (680 kg/h), preconditioner temperature (88–89°C), and extruder screw speed (435 RPM). Variable inputs were controlled by the operator and were recorded at three timepoints for each parameter. Extruder mass flow rate was measured, but not recorded, by collecting material out of the extruder into a bucket for 1 min and product bulk density was measured using a 1‐L cup. As this was an observational study and not a processing study, rated screw speed of the extruder, rated motor load, and no‐load torque were not recorded at the time of production, and thus, specific mechanical energy was unable to be calculated. In‐barrel moisture could not be calculated as the rate of steam injected in the extruder could not be measured due to available equipment limitations as this was also not within the scope of the original study.

**TABLE 3:** Processing parameters of the test diets with different primary protein sources.

Item	Diet^a^		
	BAS	CHK	PEA
Raw material			
Dry feed rate, kg/h	680.4	680.4	680.4
Preconditioner			
Temperature,°C	89	88	88
Steam injection, kg/h	53	57	52
Water injection, kg/h	109	109	95
Extruder			
Water injection, kg/h	0	0	0
Barrel steam	No	Yes	Yes
Extruder screw speed, RPM	435.4	435.4	435.4
Knife speed, RPM	1003	1003	1092
Motor load, A	83.6	77.6	62.5
Dryer			
Temperature,°C	121	121	121
Top bed residence time, min	19	19	19
Bottom bed residence time, min	30	30	30

Abbreviations: BAS, basal diet containing lamb as the primary protein source; CHK, chicken diet containing chicken and lamb as the primary protein sources; PEA, pea diet containing peas and lamb as the primary protein sources.

^a^ All diets produced by The J.M. Smucker Co. (Orrville, Ohio, USA) in their pilot plant in Topeka, Kansas, USA.

The kibbles were dried in a double‐pass oven drier (Series 4800, Wenger Manufacturing, Sabetha, KS) at 121°C for 19 min on the first pass and 30 minutes on the second pass targeting a moisture content between 8% and 9%. Moisture content of the kibble off the drier was measured using near‐infrared spectroscopy (**NIRS**). Dried product was coated with chicken fat, liquid palatant, and animal digest according to the diet formulations (Table 1). Once coated, diets were bagged and heat sealed into white opaque plastic bags and prepared for shipment to the University of Guelph, Guelph, Ontario, Canada.

All three diets were shipped from the processing facility in Topeka, Kansas, USA, on the same day and under the same shipping conditions. Diets arrived in sealed opaque plastic bags at the Central Animal Facility (University of Guelph, Guelph, ON, Canada) in February 2021 and were stored on pallets in the feed storage room at a temperature and humidity of approximately 19–20°C and 60%–65%, respectively, for 7 months (February–September 2021). During this time, all diets remained in the sealed opaque plastic bags, and none were moved. In September 2021, opened bags of the diets were transferred to black opaque 52 L airtight pet food container bins and stored at the same temperature and humidity as mentioned above until March 2022. The black opaque pet food containers were sterilized using an industrial animal cage washer prior to use to ensure the bins were not a source of mold contamination. Opened bags were transferred to airtight bins to minimize oxidative nutrient changes due to exposure to room air and light and mimic the environment of pet foods stored in sealed bags. As expiry dates of commercially available dry pet foods are typically set at 12–18 months post‐production from the manufacturer, the projected expiry date of all three experimental diets was set at January 2022, 12 months after production.

### 2.3 Sample preparation and chemical analysis

After all diets were processed in January 2021, subsamples were taken after coating and drying and sent for nutrient analysis at ISO/IEC‐accredited Eurofins Microbiology Laboratories (Madison, Wisconsin, USA). At this time, samples of the BAS, CHK, and PEA diets were analyzed for DM, all amino acids (**AA**), all fatty acids and fat contents, and all vitamins (A, D, E, Thiamine (**B1**), Riboflavin (**B2**), Niacin (**B3**), Pantothenic acid (**B5**), Pyridoxine (**B6**), Folic Acid (**B7**), Cobalamin (**B12**)), and Choline using ISO/IEC‐approved methods.

**FIGURE 1:**
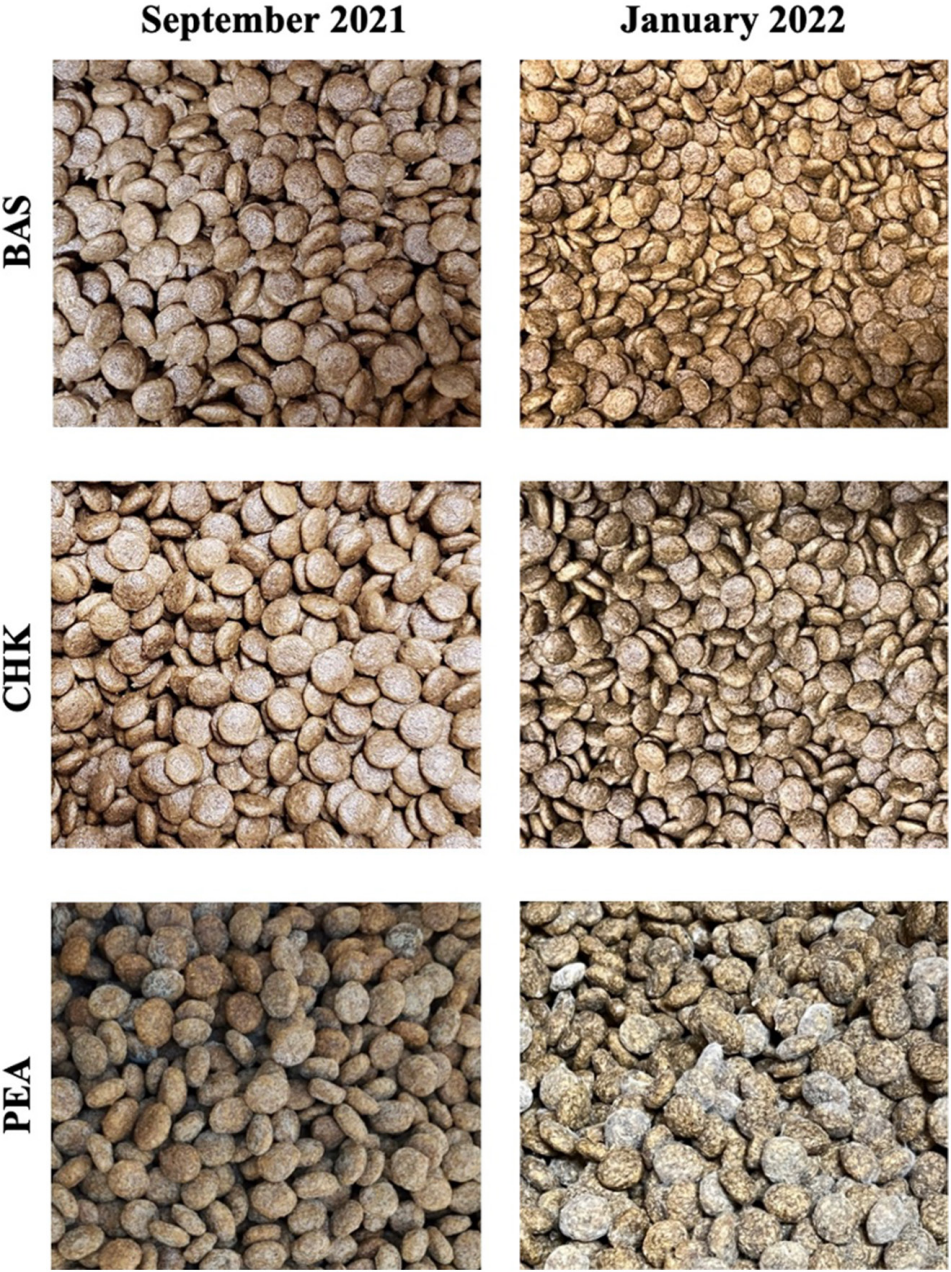
The PEA diet, but not BAS or CHK diets, shows visible mold contamination in September 2021 and at expiry in January 2022. All diets produced by The J.M. Smucker Co. (Orrville, Ohio, USA) in their pilot plant in Topeka, Kansas, USA. BAS, basal diet containing lamb as the primary protein source; CHK, chicken diet containing chicken and lamb as the primary protein sources; PEA, pea diet containing peas and lamb as the primary protein sources.

When bags of the BAS, CHK, and PEA diets were moved into opaque plastic bins in September 2021, all eight bags of the PEA diet were opened and found to contain visible mold growth upon visual and olfactory inspection. Eight bags of each of the BAS and CHK diets were also opened and inspected; however, visual mold contamination was not found in these diets (Figure 1). To explore this, a minimum of 10 subsamples per bin were taken randomly and from all areas of each bin to ensure homogeneity, and in September 2021, all test diets were sent for analysis of mycotoxin concentrations and water activity (**a**_**w**_), as well as mold speciation identification. These analyses were conducted by two laboratories to account for laboratory and methodological variation of reported results for whichever analyses the laboratory provided within these parameters.

Samples sent to Agriculture and Food Laboratory (AFL; University of Guelph, Guelph, ON, Canada) in September 2021 were analyzed for their complete mycotoxin panel including total aflatoxin (B_1_, B_2_, G_1_, and G_2_), deoxynivalenol (**DON**), fumonisin, total ochratoxin (A and B), T‐2 and HT‐2 as a total, and zearalenone using commercially available ELISA test kits and water activity using method MFLP‐66 (Health Canada, 2020). The lowest detectable limits for the detection of total aflatoxins, DON, and total ochratoxin were 1.0, 25.0, and 2.0 ppb, respectively. Microbial species identification was conducted by first isolating genomic DNA from the microorganisms and amplified using polymerase chain reaction (**PCR**), and the PCR product (16S/18S or ITS regions) size was confirmed by gel electrophoresis, purified, and sequenced using ABI 3730 XL Genetic Analyzer (Applied Biosystems). The sequences were analyzed using the ABI PrismTM Sequencing Analysis software to obtain a high‐quality consensus sequence for each sample. The sequence was then aligned with published sequences in the NCBI database using the BlastN software (www.ncbi.nlm.nih.gov) to determine the best similar species.

Samples sent to Activation Laboratories Ltd. (ACTlabs; Ancaster, ON, Canada) in September 2021 were analyzed for their complete mycotoxin panel including aflatoxin B_1_, B_2_, G_1_, and G_2_, DON, 3‐Acetyl‐deoxynivalenol, 15‐Acetyl‐deoxynivalenol, fumonisin B_1_, and B_2_, ochratoxin A, sterigmatocystin, T‐2, HT‐2, zearalenone, and mycophenolic acid using an in house proprietary method which utilized an organic solvent extraction followed by analysis using liquid chromatography–mass spectrometry/mass spectrometry technology, in addition to water activity using method 1241 (USP, 2010). The lowest detectable limits for the detection of both total and individual aflatoxins, DON, ochratoxin, and mycophenolic acid were 1.0, 60.0, 3.0, and 30 ppb, respectively.

To compare mold and subsequent mycotoxin production of the diets over time, samples of the BAS, CHK, and PEA diets were sent again in January 2022 (at their expiry) to ACTlabs for their complete mycotoxin panel and water activity using the same sampling methods as outlined above and to AFL for their complete mycotoxin panel, mold speciation, and mold count analysis using method MFHPB‐22 (Health Canada, 2018).

After receiving results from both AFL and ACTlabs in January 2022, analyzing the BAS, CHK, and PEA diets for potential changes in nutrient contents over time became of interest in order to determine if mold contamination might impact these parameters. In March 2022, samples of the BAS, CHK, and PEA diets were sent to ISO/IEC‐accredited Eurofins Experchem Laboratories Inc. (North York, Ontario, Canada). Samples were sent to Eurofins Experchem Laboratories Inc. (North York, Ontario, Canada) instead of Eurofins Microbiology Laboratories (Madison, Wisconsin, USA) to ensure that shipping time and conditions to the laboratory would be consistent. Both laboratories used the same methodologies to minimize inter‐laboratory variation.

### 2.4 Statistical analysis

Water activity was analyzed in September 2021 from both AFL and ACTlabs. Therefore, statistical analysis of water activity from the September 2021 sampling period was conducted using the GLIMMIX procedure of SAS (SAS Inst. Inc., Cary, NC) with diet as fixed effect and lab as a random effect. Differences among individual means were assessed using the Tukey–Kramer posthoc test. A probability of less than or equal to .05 was considered significant and *p* > .05 was considered not significant. All other chemical analyses were performed on one sample per diet per timepoint so statistical comparisons could not be made on those parameters.

## 3 RESULTS

### 3.1 Processing parameters

Similar wet bulk density out of the extruder was achieved through adjustment of the following parameters: steam injections in the preconditioner for the PEA and BAS diets were 9% and 7% less, respectively, than the CHK diet. Water injection in the preconditioner for the PEA diet was 13% less than both the BAS and CHK diets, and barrel steam in the extruder was only used for the CHK and PEA diets. Knife speed in the extruder was 8% faster for the PEA diet than both the BAS and CHK diets. Motor load in the extruder was greatest in the BAS diet (83.6 A) and was 7% and 25% less than in the CHK and PEA diets, respectively (Table 3). All other processing parameters were kept as set. At the time of production, moisture content of all diets off the dryer measured by NIRS did not differ from the target moisture content of 8%–9%. However, when DM of all diets was determined after coating using AOAC method 930.15 (AOAC, 2005), the moisture contents of the PEA and CHK diets were 38% and 20% greater than the BAS diet, respectively (Table 3).

### 3.2 Water activity, mycotoxin, and mold contamination

Water activity of samples analyzed in September 2021 was approximately 26% greater in the PEA diet than the BAS diet (*p* < .05) but neither differed from the CHK diet (Table 4). Samples analyzed in January 2022 showed a similar pattern of a_w_ as those observed in September 2021 but were on average 15% lower.

**TABLE 4:** The water activity (a_w_), mycotoxin, and mold contamination in experimental diets sampled in September 2021 and January 2022.

Item	Diet^a^						SEM^b^	*p*‐value
	BAS September 21	BAS January 22	CHK September 21	CHK January 22	PEA September 21	PEA January 22		
Water Activity, a_w_ ^c^	0.55^b^	0.46	0.63^a,b^	0.54	0.74^a^	0.64	0.03	0.039
Mycotoxin^d^								
DON, ppb^e^	84.1	81.7	88.0	84.0	105.2	87.6	–	–
Total aflatoxin, ppb^e^	–	–	–	–	2.1	–	–	–
Total ochratoxin, ppb^e^	–	–	–	–	3.3	3.4	–	–
Mycophenolic acid, ppm^f^	0.06	0.10	0.07	0.07	–	–	–	–
Mold count, cfu/g	–	<5.0	–	<5.0	–	14,500		
Microbiological species detected^g^								
Total no. detected	3	0	0	3	5	3	–	–
*Aspergillus* spp.	1 species	ND	ND	ND	2 species	3 species	–	–
*Penicillium* spp.	ND	ND	ND	ND	1 species	ND	–	–
*Kocuria marina*	ND	ND	ND	ND	1 species	ND	–	–
*Acinetobacter lwoffii* or *Prolinoborus fasciculus*	ND	ND	ND	ND	1 species	ND	–	–
*Paenibacillus* spp.	1 species	ND	ND	ND	ND	ND	–	–
*Bacillus* spp.	1 species	ND	ND	1 species	ND	ND	–	–
*Staphylococcus capitis* or *Staphylococcus caprae*	ND	ND	ND	1 species	ND	ND		
*Rhodotorula mucilaginosa* or *Rhodotorula alborubescens*	ND	ND	ND	1 species	ND	ND		

*Note*: ^a,b^Values with different letters within the same row differ (*p* < .05).

^a^ All diets produced by The J.M. Smucker Co. (Orrville, Ohio, USA) in their pilot plant in Topeka, Kansas, USA. BAS = Basal diet containing lamb as the primary protein source; CHK = Chicken diet containing chicken and lamb as the primary protein sources; PEA = Pea diet containing peas and lamb as the primary protein sources. September 21 = indicates results from September 2021 sampling timepoint; January 22 = indicates results from January 2022 sampling timepoint.

^b^ Maximum value of standard error of the means.

^c^ Water activity (a_w_) was analyzed at two different labs (Agriculture and Food Laboratory (Guelph, ON, Canada), and Activation Laboratories (Ancaster, ON, Canada)) for samples collected in September 2021; however, water activity was only analyzed at one lab (Activation Laboratories (Ancaster, ON, Canada)) in January 2022. Statistical comparisons made are only for samples collected in September 2021; n = 2.

^d^ Only mycotoxins detected presented.

^e^ Mycotoxin analyzed using ELISA by Agriculture and Food Laboratory (Guelph, ON, Canada).

^f^ Mycotoxin analyzed using LC–MS/MS technology by Activation Laboratories (Ancaster, ON, Canada).

^g^ Contamination levels not given. “ND” indicates no species were detected. Microbial species ID determined by 16S/18S rRNA gene methodology by Agriculture and Food Laboratory (Guelph, ON, Canada).

Deoxynivalenol was detected in all diets at both the September 2021 and January 2022 sampling periods at similar levels (approximately 85 ppb; Table 4). However, DON levels were found to be approximately 18% greater in the PEA diet sampled in September. Total aflatoxin was only detected in the PEA diet at the September 2021 sampling period, while total ochratoxin was only detected in the PEA diet but at both sampling times. Mycophenolic acid was detected in both September 2021 and January 2022 in both the BAS and CHK diets, but not the PEA diet. Mycophenolic acid contamination in the BAS diet increased by 40% between September and January; however, it remained constant in the CHK diet at both sampling timepoints.

The BAS diet had detectable levels of one species of each *Aspergillus*, *Paenibacillus*, and *Bacillus* spp. at the September 2021 sampling period. However, no mold species were detected in the BAS diet at the January 2022 sampling period and mold counts of the BAS diet were below the detectable level at this timepoint (<5.0 cfu/g). The CHK diet only had detectable levels of one species of each *Bacillus* spp., *Staphylococcus capitis* or *Staphylococcus caprae*, and *Rhodotorula mucilaginosa* or *Rhodotorula alborubescens* at the January 2022 sampling period; however, mold counts of the CHK diet at this timepoint were below detectable levels (<5.0 cfu/g). At the September 2021 sampling timepoint, the PEA diet had two species of *Aspergillus* spp. detected in addition to one species of each *Penicillium* spp., *Kocuria marina*, and *Acinetobacter lwoffii* or *Prolinoborus fasciculus*. At the January 2022 sampling timepoint, only three species of *Aspergillus* spp. were detected, although mold count analyses of the PEA diet at this timepoint showed contamination of 14,500 cfu/g.

### 3.3 Nutrient analyses between January 2021 and March 2022

The AA contents of all diets sampled in January 2021 and March 2022 generally stayed within 10% difference from each other between sampling timepoints, except for Met which decreased by 14% in the CHK diet but not the BAS and PEA diets, cystine (**Cys**) which decreased in the CHK and PEA diets by 15% and 20%, respectively, but not in the BAS diet, tyrosine (**Tyr**) which decreased by 30%, 25%, and 27% across the BAS, CHK, and PEA diets, respectively, and taurine (**Tau**) which decreased by 50%, 42%, and 55% across the BAS, CHK, and PEA diets, respectively (Table 5).

**TABLE 5:** Change in analyzed amino acid, fatty acid, and vitamin contents of the BAS, CHK, and PEA diets at processing in January 2021 and 2 months after expiry in March 2022 on a dry matter basis.

Item	Diet^a^					
	BAS January 21^b^	BAS March 22^c^	CHK January 21	CHK March 22	PEA January 21	PEA March 22
Indispensable AA, %						
Arg	1.02	0.91	1.38	1.23	1.77	1.65
His	0.29	0.32	0.41	0.43	0.50	0.53
Ile	0.51	0.51	0.72	0.71	0.90	0.90
Leu	1.09	1.08	1.44	1.44	1.74	1.74
Lys	0.77	0.83	1.13	1.20	1.44	1.53
Met	0.27	0.27	0.37	0.32	0.33	0.32
Phe	0.61	0.61	0.80	0.81	1.05	1.05
Thr	0.60	0.60	0.78	0.80	0.92	0.93
Trp	0.15	0.16	0.20	0.22	0.25	0.26
Val	0.75	0.81	0.98	1.01	1.16	1.21
Dispensable AA, %						
Ala	1.00	0.96	1.30	1.31	1.33	1.33
Asp	1.18	1.20	1.62	1.64	2.23	2.24
Cystine	0.20	0.19	0.25	0.21	0.32	0.26
Tyr	0.47	0.33	0.61	0.45	0.80	0.62
Glu	2.26	2.22	2.90	2.91	3.73	3.79
Gly	1.47	1.47	1.81	1.90	1.77	1.83
Pro	1.21	1.20	1.46	1.41	1.54	1.50
Ser	0.67	0.67	0.84	0.86	1.07	1.10
Tau	0.062	0.030	0.078	0.045	0.058	0.026
Fatty acids, %						
Caprylic (C8:0)	0.008	0	0	0	0	0
Capric (C10:0)	0	0	0.008	0	0	0
Lauric (C12:0)	0.012	0	0.013	0	0.010	0
Myristic (C14:0)	0.184	0.173	0.195	0.182	0.180	0.163
Myristoleic (C14:1)	0.017	0	0.019	0	0.021	0
Pentadecanoic (C15:0)	0.034	0	0.036	0	0.036	0
Palmitic (C16:0)	2.707	2.616	3.099	3.076	3.181	3.298
Palmitoleic (C16:1)	0.437	0.367	0.523	0.467	0.551	0.523
Margaric (C17:0)	0.083	0.072	0.088	0.074	0.089	0.077
Stearic (C18:0)	1.528	1.357	1.672	1.477	1.688	1.539
Oleic (C18:1)	3.918	3.382	4.504	4.010	4.880	4.645
Linoleic (C18:2)	1.637	1.341	1.962	1.659	2.258	2.077
Linolenic (C18:3)	0.145	0.093	0.159	0.102	0.223	0.166
Arachidic (C20:0)	0.017	0	0.020	0	0.025	0
Gondoic (C20:1)	0.036	0.020	0.042	0	0.048	0.046
Eicosadienoic n‐6 (C20:2)	0.015	0	0.019	0	0.021	0
Arachidonic (C20:4)	0.034	0.024	0.052	0.038	0.039	0.035
Eicosapentaenoic (C20:5)	0.015	0	0.013	0	0.017	0
Behenic (C22:0)	0	0	0.009	0	0.009	0
Docosatetraenoic (C22:4n‐6)	0.010	0	0.013	0	0.010	0
Docosapentaenoic (C22:5) (DPA)	0.016	0	0.018	0	0.017	0
Docosahexaenoic (C22:6) (DHA)	0.010	0	0.009	0	0.008	0
Lignoceric (C24:0)	0.008	0	0.010	0	0.010	0
Saturated FA	4.37	4.22	4.92	4.80	4.99	5.08
Monounsaturated fatty acids	4.65	3.77	5.34	4.51	5.74	5.21
Polyunsaturated fatty acids	1.82	1.45	2.17	1.80	2.51	2.29
Omega‐3 fatty acids	0.19	0.09	0.20	0.10	0.26	0.17
Omega‐6 fatty acids	1.71	1.37	2.07	1.69	2.35	2.13
Total fatty acids	11.35	9.67	13.04	11.33	13.80	12.80
Total Fat	10.79	10.12	12.26	11.86	13.80	13.39
Vitamins						
Vitamin A, IU/kg	10106.96	5370.82	10657.75	7594.38	14367.16	13111.79
Vitamin D, IU/kg	709.45	415.66	781.49	543.56	941.85	643.16
Vitamin E, IU/kg	96.16	141.11	114.83	151.57	144.81	165.03
Thiamine, mg/kg	14.16	18.60	15.38	17.49	16.99	19.22
Riboflavin, mg/kg	19.97	9.77	15.94	9.91	19.73	11.02
Niacin, mg/kg	168.09	143.29	181.72	156.09	179.02	153.72
Pantothenic, mg/kg	33.40	16.85	33.44	17.60	31.47	20.91
Pyridoxine, mg/kg	15.83	11.16	15.94	10.40	14.25	10.22
Folic Acid, mg/kg	4.17	4.23	3.82	3.74	3.75	3.72
B12, mg/kg	0.074	0.073	0.077	0.070	0.074	0.068
Choline, mg/kg	1866.40	2023.63	1861.76	2081.26	2143.67	2283.26

Abbreviations: BAS, basal diet containing lamb as the primary protein source; CHK, chicken diet containing chicken and lamb as the primary protein sources; January 21, indicates results from January 2021 sampling timepoint; March 22, indicates results from March 2022 sampling timepoint; PEA, pea diet containing peas and lamb as the primary protein sources.

^a^ All diets produced by The J.M. Smucker Co. (Orrville, Ohio, USA) in their pilot plant in Topeka, Kansas, USA.

^b^ Diets analyzed at Eurofins Experchem Laboratories (Madison, Wisconsin, USA).

^c^ Diets analyzed at Eurofins Experchem Laboratories (North York, ON, Canada).

The fatty acid contents of all diets sampled in January 2021 and March 2022 generally decreased between sampling timepoints, except for palmitic acid (**C16:0**) which increased by 4% in the PEA diet, but not the BAS or CHK diets, which led to a 2% increase in saturated fatty acid content of the PEA diet across sampling timepoints.

The vitamin contents of all diets sampled in January 2021 and March 2022 generally decreased between sampling timepoints, except for vitamin E which increased by 47%, 32%, and 14% in the BAS, CHK, and PEA diets, respectively, thiamine which increased by 30%, 14%, and 13% in the BAS, CHK, and PEA diets, respectively, and choline which increased by 8%, 12%, and 6% in the BAS, CHK, and PEA diets, respectively. Folic acid was the only vitamin that remained constant across all diets at both timepoints.

## 4 DISCUSSION

The goal of this observational study was twofold: (1) to quantify and determine the level of mold and mycotoxin contamination of an extruded diet intended for dogs containing peas at an inclusion level of greater than 40% (as‐fed) compared to two other diets not containing peas processed and stored under similar conditions for 8 months, and (2) how mold contamination may impact the nutrient content of the diets outside of common shelf‐life losses when assessed post‐production and 2 months post‐expiry. The subspecies of mold found in the PEA diet were not considered to be of toxic origin but likely contributed to mycotoxin contamination of this diet compared to the BAS and CHK diets. Additionally, the mold contamination of the PEA diet was likely due to its increased moisture content after processing which was incorrectly estimated by NIRS determination of DM and should be considered in the manufacturing of high‐pulse‐containing diets. It would have been beneficial to assess the dry mixture of the PEA diet for mold and mycotoxin contamination to determine whether the peas used were initially contaminated with mold or mycotoxins or if the contamination occurred mostly due to the PEA diets’ high moisture content and a_w_. Unfortunately, this was not conducted as it was not the primary objective of the initial project. While certain subspecies of molds and mycotoxins were detected in the BAS and CHK diets, when quantified by mold count analysis, the level of mold contamination was below detectable limits when tested in March 2022 in spite of being stored under identical conditions during this time. Despite this, a decrease in certain AA (including Met, Cys, Tyr, and Tau) was observed in all diets, which could be a result of the mold contamination. However, using a control diet without mold contamination was outside of the scope of this observational study, therefore, controlled studies on the effects of mold and mycotoxin contamination of extruded pet food on AA concentrations are warranted.

### 4.1 Processing of diets and mold development

There is no regulated limit for moisture content in dry pet food as defined by AAFCO (2023). Dry pet foods typically contain between 10% and 12% moisture according to the United States Food and Drug Administration (FDA, Animal Health Literacy, 2020). Pet foods containing >12% moisture are at greater risk for mold contamination without the use of mold inhibitors (de Brito et al., 2013). Therefore, the target moisture content of all diets was set to <10% to attempt to meet this threshold. Common errors in NIRS proximate measurements of feedstuffs are lack of generated validated predictive models and inappropriate sample preparation (Buonaiuto et al., 2021; Leiva et al., 2022). The PEA diet had a nearly 45% inclusion rate of peas, and the use of a high‐fiber‐containing ingredient may have required a different predictive model to accurately measure moisture, as has been shown in forages containing high‐fiber fractions (Buonaiuto et al., 2021). Additionally, peas are known to have a high swelling power and, like tubers, require more water during extrusion processing than cereal ingredients at the same dry feed rate (Gujska et al., 1994; Pezzali & Aldrich, 2019). The amount of steam added to the PEA diet in the extruder could not be quantified, and this likely contributed to the higher moisture content of the PEA diet as the BAS, CHK, and PEA diets were dried at identical temperatures for an identical amount of time. A high a_w_, particularly between 0.60 and 0.87, often leads to increased risk of mycotoxigenic mold growth (Beuchat, 1981). The BAS diet was below this threshold in September 2021 and January 2022; the CHK diet was only within this range in September 2021 and the PEA diet was within this range during September 2021 and January 2022. The three experimental diets were not analyzed for a_w_ right after production; however, it is likely that the a_w_ of the PEA diet was also higher than the BAS and CHK diets at that time. As the diets were not expected to have a moisture content of over 12%, were intended to be fed within 2 months of the January 2021 production, and arrived in sealed bags, mold inhibitors were not added. Further, it has been proposed that keeping moisture content of extruded pet foods lower than 11.5% could also suppress fungal growth (Bueno et al., 2001). The act of extrusion and drying to <9% moisture may have reduced growth of *Aspergillus* spp. detected in BAS diet, leading to undetectable Aflatoxin and Ochratoxin levels (Martínez‐Martínez et al., 2021). While molds are susceptible to high heat during extrusion, mycotoxins are generally chemically and thermally stable, which may further explain why mycotoxins were detected in the BAS and CHK diets, but mold species were not detected between September 2021 and January 2022 (Leung et al., 2006; Mobashar, 2023). This suggests that a common ingredient was contaminated with mycotoxins prior to extrusion; however, testing raw ingredients for mycotoxin contamination was outside of the scope of the current study. Overall, it is likely that the visible mold contamination on the PEA diet was due primarily to the high moisture input in the extruder from steam and the inaccurate determination of its greater moisture content using NIRS, which led to the PEA diet having a high a_w_. This suggests that extruded diets with high pea inclusion likely require accurate predictive NIRS models, if they are being used in quality control steps during production, and extra consideration of processing parameters such as how much water and steam are being added during extrusion and drying temperature to ensure moisture contents of these diets remain less than at least 11.5%.

### 4.2 Mold and mycotoxin contamination

All diets were found to contain mold contamination; however, only the PEA diet was found to contain molds at both sampling timepoints and registered mold counts above a detectable limit when tested in January 2022 (Figure 1). The European Pet Food Industry Federation (FEDIAF, 2018) has identified several microbiological species that need to be monitored as they are likely to cause illness or deleterious health outcomes to animals such as *Enterobacteriaceae*, pathogenic *Escherichia coli*, *Salmonella*, *Staphylococcus aureus*, *Listeria monocytogenes*, *Clostridium perfringens*, *Clostridium botulinum*, *Aeromonas*, and *Campylobacter*. In addition to the above, the FDA also provides methods of detection for *Shigella*, *Yersinia enterocolitica*, *Vibrio*, and *Bacillus cereus* species in foods that should be monitored (FDA, Laboratory Methods (Food), 2022). Other molds such as *Aspergillus flavus*, *A. fumigatus*, and *A. parasiticus* are known to produce aflatoxins that can cause aflatoxicosis (Amaike & Keller, 2011). *Aspergillus niger*, *A. ochraceus*, and *Penicillium verrucosum* molds can produce ochratoxin A and *Fusarium* spp. can produce a variety of mycotoxins including DON (Leung et al., 2006; Schuster et al., 2002). Currently, there are no defined recommendations for “safe” mold counts in pet foods; however, all diet bags were stored properly and remained unopened from production until discovery of mold on the PEA diet in September 2021, but something different may have been observed if diets were exposed to higher humidity or temperature. Despite this, none of the microbiological species considered problematic by FEDIAF (2018) and FDA, Laboratory Methods (Food) (2022) were detected in the PEA diet, and it is unlikely that the species of *Aspergillus*, *Penicillium*, or *Bacillus* molds were of concern as there was no marked increase in mycotoxin production between the two sampling points.

While all diets contained a variety of different mycotoxins, none were greater than the most current regulatory guidance (EUL, 2016; FDA, Centre for Veterinary Medicine, 2010, 2019). Deoxynivalenol is produced by *Fusarium* spp. so the presence of DON in all diets at both sampling timepoints suggests there was previous *Fusarium* spp. contamination and thus low‐level DON contamination in one or more common raw ingredients, such as barley, prior to processing, as there was no *Fusarium* spp. detected when diets were analyzed after processing (Leung et al., 2006; Scott, 1997; Trucksess et al., 1995). The highest level of DON reported was in the PEA diet at the September 2021 sampling which was nearly 48 times less than the FDA guidance. Aflatoxins are primarily produced by *Aspergillus* spp. (Moss, 1996), which were detected in the PEA diet. Aflatoxins are known to be carcinogenic and hepatotoxic with aflatoxin B_1_ being the most toxic (Puschner, 2002). Böhm and Razzai‐Fazeli (2005) observed that dogs consuming diets containing 0.05–0.3 mg of aflatoxin per kg feed displayed anorexia, lethargy, jaundice, disseminated intravascular coagulation, and death when consuming aflatoxin at these levels over 6–8 weeks. However, the level of aflatoxin found in the PEA diet in September 2021 was approximately 24 times less than the minimum dose that Böhm and Razzai‐Fazeli (2005) reported and nearly 10 times less than the FDA advisement. Ochratoxins are produced by both *Aspergillus* spp. and *Penicllium* spp. (Leung et al., 2006), which were both detected in the PEA diet. Ochratoxins are divided into four homologs (A, B, C, and D); however, ochratoxin A is considered the most prevalent and ochratoxins A and C are the most toxic (Haschek et al., 2002). However, there is limited research on the toxic or lethal dose of ochratoxin A in dogs (Puschner, 2002). According to European Union Law (EUL), the guidance level for ochratoxin A is 0.01 mg/kg in dog and cat foods with 12% moisture (EUL, 2016); however, at this time no other regulatory agency has defined an actionable level of ochratoxin A in pet food. The level of total ochratoxin detected in the PEA diet was approximately 3 times less (0.003 mg/kg of feed at 12.3% moisture) than the EUL guidance level, which indicates that even without a clearly defined toxic dose defined for dogs, that the contamination of total ochratoxin in the PEA diet would not have been expected to cause deleterious health outcomes for dogs. Mycophenolic acid is typically produced by *Penicillium stoniferum* and while it is considered a mycotoxin, due to its immunosuppressive action, pharmaceutical formulations of mycophenolic acid (e.g., mycophenolate mofetil; **MMF**) have been utilized in human medicine since the 1990s due to its immunosuppressive action (Klotsman et al., 2019). Currently, there is no actionable limit for mycophenolic acid in pet foods defined by regulatory agencies. Mycophenolic acid was not detected in the PEA diet but was detected in the BAS and CHK diets at levels ranging from 0.06 to 0.10 mg/kg of feed. As no *Penicillium* spp. were detected in the BAS and CHK diets at both sampling timepoints, it is likely there was a low level of mycophenolic acid contamination in the corn starch used prior to processing, which was the only ingredient common to both the BAS and CHK diets and not present in the PEA diet (Gruber‐Dorninger et al., 2017). These levels are unlikely to cause deleterious health outcomes and are of little concern. Overall, while mold and mycotoxin contamination were found, in all cases this contamination did not exceed levels known to cause physiological consequences, but it is important to note that all diets were stored properly and in climate‐controlled conditions.

Practically, it is important to note that inaccurate sampling is generally considered the biggest source of error in mycotoxin analysis as mycotoxins and molds are not evenly distributed throughout feed (Leung et al., 2006). All test diets sent for mycotoxin and mold analysis were subsampled with a minimum of 10 samples per bin from all areas to ensure as homogeneous a sample as possible; however, despite our best efforts, sampling error may have contributed to the decrease in DON contamination found in the PEA diet between the September 2021 and January 2022 samplings. Further, the analytical method used to detect mycotoxins may contribute variation to mycotoxin quantification, although less than from sampling (Leung et al., 2006). Of these, liquid chromatography–mass spectrometry/mass spectrometry (**LC‐MS/MS**) is generally regarded to be one of the most sensitive methods allowing for the identification of conjugated mycotoxins and generally allowing for much lower detection limits than with other analyses such as enzyme‐linked immunosorbent assay (**ELISA**) which is why both methods were used in this study (Leung et al., 2006; Tansakul et al., 2013). Overall, despite levels of DON, total aflatoxin, total ochratoxin, and mycophenolic acid mycotoxins being detected in all test diets, they were not considered to be toxic for dogs in this observational study. Mold and mycotoxin contamination can be impacted by both increased humidity and temperature during storage which emphasizes the importance of proper testing of raw ingredients prior to processing and storage of pet foods by consumers to prevent increased contamination that may lead to mycotoxicosis in dogs.

### 4.3 Amino acid, fatty acid, and vitamin contents

During processing, some destruction of amino acids is known to occur (e.g., Maillard reaction products), with the greatest losses in standardized ileal digestibilities found in lysine, threonine, and aspartate in extruded diets compared to more mildly processed diets such as mildly cooked fresh or freeze‐dried pet foods (Geary et al., 2023). But generally, processing improves amino acid digestibility and availability, particularly in pulse ingredients (Cargo‐Froom et al., 2023; Geary et al., 2023). During storage, the crude protein and amino acid content of feeds generally remain stable (Bartov et al., 1982). This is in agreement with our results which found that concentrations of all AA other than Met, Cys, Tyr, and Tau remained relatively stable across all diets and sampling timepoints indicating that both Eurofins labs (Eurofins Experchem Laboratories Inc. and Eurofins Microbiology Laboratories) produced repeatable results for analysis of AA. There is a dearth of research exploring the effect of storage of pet foods contaminated with even low levels of mold on dietary AA concentrations; however, the decrease in these AA may be due to several factors. Liu et al. (2019) found that peanuts stored at 25°C for 320 days had lower concentrations of free Met, Cys, and Tyr due to increased lipid oxidation during storage. Measuring lipid oxidation was outside of the scope of this observational study; however, it is likely that storage of these diets contributed to this effect particularly in the BAS and CHK diets which had no visible mold contamination. Additionally, it is possible that mold contamination may have played a role in the decrease in Met, Cys, Tyr, and Tau in the PEA diet. Megalla et al. (1990) found that certain *Aspergillus* and *Penicillium* spp. exhibited some of the highest proteolytic enzyme activity, particularly for feed AA. This may suggest that other mycotoxigenic mold subspecies may possess proteolytic activity for certain AA and lead to reduced AA concentrations available to the animal in conjunction with storage losses.

Over the last several years, there has been a growing concern of the adequate provision of sulfur‐containing AA Met, Cys, and Tau and their potential effect on the increased prevalence of cases of nutritionally mediated dilated cardiomyopathy (**DCM**) in dogs (Mansilla et al., 2019). As we saw a decrease in Met in the CHK diet, Cys in both the CHK and PEA diets, and Tau across all diets this emphasizes the need for more research into how storage and mycotoxigenic molds may also affect the availability of certain AA in canine diets. It is important that pet food companies and retailers consider robust shelf stability studies, whether accelerated or not, under a variety of shipping conditions and storage conditions throughout the entire life of the product to ensure that the provisions of Met, Cys, and Tau are sufficient to prevent nutritional deficiencies that may contribute to the development of DCM. Conducting these studies and sharing results through publicly available scientific literature can help guide the safe development of pet foods and build on regulations for pet foods.

Nutrients such as fatty acids and vitamins are known to degrade in extruded pet foods over the shelf‐life of the product (Coelho, 1991; Hołda & Głogowski, 2016); however, foods must meet the American Association of Feed Control Officials (Association of American Feed Control Officials (AAFCO), 2023) nutrient density recommendations at the end of shelf‐life using accelerated environmental chambers. This was observed for all fatty acids, overall fat content, and for the majority of vitamins across all diets except for an increase in C16:0 in the PEA diet leading to a subsequent increase in the saturated fatty acid content of the PEA diet and increases in vitamin E, thiamine, and choline across all diets between January 2021 and March 2022, respectively. In terms of C16:0, while some of this effect may be attributed to inter‐lab variation, it is possible that this may be due to the increased number of *Aspergillus* spp. present in the PEA diet, which increased from two to three between the September 2021 and January 2022 sampling timepoints. One of the primary fatty acids found in the cell membrane of *Aspergillus oryzae* is C16:0 (Ma et al., 2019). Unfortunately, mold count analysis was not conducted during the September 2021 sampling, which may have provided evidence for this effect. However, visual inspection of the PEA diet between the September 2021 and January 2022 indicated an overall increase in mold contamination of this diet, which was not observed in the BAS or CHK diets and confirmed by the mold count analysis. In terms of vitamin content, the nutrient analysis results for vitamin E from March 2022 reported vitamin E in mg/kg, and so to allow for an accurate comparison to results from January 2021 this value was converted to IU/kg. To complete this conversion, it was assumed that all vitamin E provided was only of synthetic origin when there were natural sources of vitamin E present in ingredients; however, this proportion was unable to be quantified. Therefore, some error may have been introduced through this assumption. However, vitamin E is relatively stable when exposed to processing conditions and like folic acid is mainly susceptible to degradation when exposed to oxidation and UV light that could occur during storage (Coelho, 1991; Riaz et al., 2009). As all diets were stored in identical conditions, this result is expected. Concentrations of choline increased over time; however, considering the stability of choline chloride during storage of feed, this observation is not surprising (Cooley & Christiansen, 1948; Yang et al., 2019). Surprisingly, thiamine concentrations in all diets increased over time. Thiamine degradation in the processing and storage of pet food is well researched as thiamine is a labile vitamin sensitive to many processing conditions (DiSabatino et al., 2021). It is unknown what caused the reported increase in thiamine between both sampling timepoints for all diets as both labs used the same methodology for determination of thiamine (method 942.23; AOAC, 2005). There is a dearth of research that has quantified losses of all vitamins in feeds contaminated with mold, so it is unknown if mold contamination in the PEA diet impacted the vitamin content over the two sampling timepoints. However, as there was only one observation per diet per timepoint for nutrient analysis and the rate of decrease or increase in vitamin concentration was generally similar across all diets, it is likely that changes in vitamin concentrations of all diets were due to common losses during storage.

As this was an observational study, we were limited to one observation per sample per sampling timepoint for nutrient analysis so statistical analysis of our results, particularly for changes in nutrient content of the diets, was not appropriate and therefore conclusions should be carefully drawn. Additionally, despite both labs used for nutrient analysis of all diets at both sampling timepoints being ISO/IEC accredited and using the same methods, some variations seen between sampling timepoints may have been due to inter‐lab variation. The second sampling timepoint for nutrient analysis in March 2022 was 2 months after expiry of the diets and the second sampling for mold and mycotoxin contamination. Therefore, the mold and mycotoxin contamination of the diets may have further changed by that point. This observational study still provides valuable insights into the potential effects of extruded pet food with different complex matrices on mold growth and nutrient retention over time and may serve as a starting point for future studies. The authors recommend repeated and more frequent sampling of diets for both molds, mycotoxins, and nutrient content under a variety of storage conditions in future research. These will help mimic the conditions found in consumers' homes and would allow for meaningful statistical comparisons to be made in addition to providing greater insight into how this may impact nutrient availability in pet foods intended for dogs.

## 5 CONCLUSION AND IMPLICATIONS

While mycotoxin levels were not at dangerous levels for dogs and molds were not of toxic origin, the PEA diet was contaminated with both molds and mycotoxins at both sampling timepoints. Mold contamination of the PEA diet was likely due to the high moisture content and high a_w_ of the PEA diet after processing which was missed due to the incorrect determination of its moisture content using NIRS. Additionally, other characteristics of peas such as their high swelling power which required more steam input in the extruder to maintain the same wet bulk density likely contributed to the higher moisture content of the PEA diet. Mold development in the BAS and CHK diets was likely limited due to processing that resulted in a low moisture content. Storage of extruded diets and mold development may also negatively impact the availability of nutrients that may put dogs at risk for nutrient deficiencies if not closely monitored. This emphasizes the need for robust quality control measures during processing and comprehensive shelf‐life studies to ensure the safety of dog diets with different ingredients. Dog diets containing peas, and other ingredients that behave similarly to peas during extrusion (potentially also when these diets also contain certain grains susceptible to mycotoxin contamination), likely require greater attention to certain processing parameters such as longer drying time/higher drying temperatures compared to diets without these ingredients. More research should be done to define these specific processing parameters required in order to prevent contamination and to ensure minimal nutrient damage to promote the health of dogs consuming extruded diets containing peas.

## AUTHOR CONTRIBUTIONS

**Michelina Crosbie:** Conceptualization (equal); formal analysis (lead); investigation (lead); methodology (lead); visualization (lead); writing – original draft (lead); writing – review and editing (equal). **Julia G. Pezzali:** Investigation (supporting); supervision (supporting); writing – review and editing (equal). **Leslie Hancock‐Monroe:** Conceptualization (equal); funding acquisition (equal); investigation (supporting); methodology (supporting); resources (lead); supervision (lead); writing – review and editing (supporting). **Preston R. Buff:** Investigation (supporting); resources (supporting); supervision (supporting); writing – review and editing (supporting). **Anna K. Shoveller:** Conceptualization (equal); funding acquisition (equal); investigation (supporting); methodology (equal); supervision (lead); writing – review and editing (supporting).

## FUNDING INFORMATION

Mitacs and The J.M. Smucker Co. (Orrville, Ohio, USA).

## CONFLICT OF INTEREST STATEMENT

The authors M.C., J.G.P, L.H‐M, and P.R.B declare that they have no conflicts of interest. A.K.S. declares that she is the Champion Petfoods Chair in Canine and Feline Nutrition, Physiology, and Metabolism and consults for Champion Petfoods. A.K.S and has received honoraria and research funding from various pet food manufacturers and ingredient suppliers.

## ETHICS STATEMENT

This study does not involve any human or animal testing.

## Data Availability

All data is available in the presented manuscript.
